# Micronutrient dose response (MiNDR) study among women of reproductive age and pregnant women in rural Bangladesh: study protocol for double-blind, randomised, controlled trials

**DOI:** 10.1136/bmjopen-2024-090108

**Published:** 2025-01-04

**Authors:** Towfida Jahan Siddiqua, Kerry J Schulze, SM Tafsir Hasan, Khalid Bin Ahsan, Sulagna Bandyopadhyay, Eleonor Zavala, Hasmot Ali, Rezwanul Haque, Hasan Mahmud Sujan, Md. Hafizur Rahman, Sarah Baker, Katherine K Stephenson, Ximing Ge, Ethan K Gough, Brooke Langevin, Lee Shu Fune Wu, Brian Dyer, Anjan Kumar Roy, Mohammad Jubair, Amena Al Nishan, Michael Rosenblum, Mathangi Gopalakrishnan, Klaus Kraemer, Daniel J Erchick, Tahmeed Ahmed, Parul Christian

**Affiliations:** 1 JiVitA Project, Rangpur, Bangladesh; 2 Center for Human Nutrition, Department of International Health, Johns Hopkins Bloomberg School of Public Health, Baltimore, Maryland, USA; 3 Nutrition Research Division, International Centre for Diarrhoeal Disease Research Bangladesh, Dhaka, Bangladesh; 4 Center for Translational Medicine, University of Maryland School of Pharmacy, Baltimore, Maryland, USA; 5 Infectious Disease Division, International Centre for Diarrhoeal Disease Research Bangladesh, Dhaka, Bangladesh; 6 Department of Biostatistics, Johns Hopkins Bloomberg School of Public Health, Baltimore, Maryland, USA; 7 Office of Executive Director, International Centre for Diarrhoeal Disease Research Bangladesh, Dhaka, Bangladesh

**Keywords:** Multiple micronutrient supplementation (MMS), balanced energy and protein (BEP), dose response, micronutrient deficiencies, pregnancy nutrition Bangladesh

## Abstract

**Introduction:**

Optimising the micronutrient status of women before and during reproduction confers benefits to them and their offspring. Antenatal multiple micronutrient supplements (MMS), given as a daily tablet with nutrients at ~1 recommended dietary allowance (RDA) or adequate intake (AI) reduces adverse birth outcomes. However, at this dosage, MMS may not fully address micronutrient deficiencies in settings with chronically inadequate diets and infection. A bioefficacy study to determine amounts required to attain nutrient adequacy among women of reproductive age (WRA) and pregnant women (PW) aims to address this gap.

**Methods and analysis:**

Two, four-arm, dose-response trials (n=240 participants/trial) with a double-blind, individually randomised, controlled design are underway in 18–35 year-old WRA and PW in rural northern Bangladesh. The trials will test dose response to four levels of 19 micronutrients from 1 RDA/AI up to ~75% of the tolerable upper intake level (UL), where applicable. These levels of micronutrients are delivered in the form of a reconstituted (in water) powdered drink, daily, including a placebo drink in the control arm, plus a fortified, balanced energy and protein (BEP) food product containing each micronutrient at ~1 RDA per serving. The supplement duration is 3 months in WRA and~6 months (until birth) in PW, who are enrolled at 12–16 weeks of pregnancy; women are randomised to one of the four arms at enrolment. Supplement consumption is directly observed by study staff and weekly side effects and adverse events are monitored. Blood and urine are collected at baseline, a midpoint, and at/near the end of supplementation, with a birth visit and postpartum biospecimen collection (post supplementation) for PW. Outcomes are biomarkers of nutrient status. Pharmacokinetic modelling will estimate micronutrient intakes at which sufficiency for each nutrient without excess is achieved. Enrolment was initiated on 22 October 2023.

**Ethics and dissemination:**

The study was approved by the Institutional Review Board of Johns Hopkins Bloomberg School of Public Health and the research and ethical review committees of icddr,b, Bangladesh. A data safety and monitoring board is in place for the study. Findings will be disseminated in peer-reviewed papers and in-country meetings.

**Trial registration number:**

NCT06081114Cite Now

**STRENGTHS AND LIMITATIONS OF THIS STUDY**

- Two double-blind, individually randomised, four-arm controlled trials of increasing levels of 19 micronutrients, will determine the minimum amount of each micronutrient at which nutritional adequacy is attained among women of reproductive age and pregnant women in a typically undernourished setting.
- Direct observation of the micronutrient supplement consumption by trained research staff through daily home visits ensures high adherence.
- The dose response will be assessed using over 25 micronutrient-specific static and functional biomarkers of nutrient status as primary outcomes. Dose response by presupplementation status will also be revealed.
- Interactions between nutrients may be difficult to ascertain.
- Robust design with sufficient statistical power has the potential to inform nutrient requirements for women of reproductive age and during pregnancy and have an impact on policies related to the nutritional support of women before and during reproduction.

## Introduction

 Improving nutritional status impacts women’s health, supports the nutritional demands of pregnancy, improves pregnancy outcomes and imparts nutrient stores to offspring in utero and through lactation.^1^ Micronutrient (commonly used term for vitamins and minerals) deficiencies and anaemia are high in women living in low- and middle-income countries (LMICs) and are linked to adverse birth outcomes and poor child growth and development.^2 3^ The most direct and causal evidence of the link between antenatal micronutrient deficiencies and adverse birth outcomes comes from trials of multiple micronutrient supplements (MMS). The most common formulation tested in trials was the United Nations International Multiple Micronutrient Antenatal Preparation (UNIMMAP), designed by expert group consensus,^4^ which contains one recommended dietary allowance (RDA) of 15 micronutrients including iron and folic acid (IFA). In a meta-analysis of 19 trials using UNIMMAP or similar formulations, MMS significantly reduced low birth weight compared with IFA alone as standard antenatal care.^5^

The RDA is the amount of a nutrient consumed daily that is needed to maintain adequate nutritional status of nearly all (97.5%) healthy and well-nourished individuals in a population group when an estimated average requirement (EAR) distribution can be established. An adequate intake (AI) recommendation is often established as an intake goal in the absence of sufficient data to determine an EAR.^6^,^10^ Finally, the tolerable upper intake level (UL) is the highest daily intake of a nutrient that is likely to pose no risk of adverse health effects for 97.5% of a healthy population. However, these Dietary Reference Intakes (collective term for EAR, RDA, AI, UL) were established for healthy North American populations. Even so, intake recommendations for women are often based on extrapolating findings from men, have often not included pregnant women (PW), and do not report or account for ethnicity.^11 12^ Data on appropriate nutrient intakes for populations in LMICs, where diets are chronically inadequate resulting in widespread deficiencies, conditions such as infections, geohelminths, inflammation and enteric dysfunction exist—and thus where nutrient requirements could be higher—are lacking. This lack of information on micronutrient status and intakes required to resolve deficiencies in LMICs was recently recognised as a data gap by the Micronutrient Data Generation Initiative, who advocate for such global population-level data collection, especially among women of reproductive age (WRA) and young children.^13^

While the 1 RDA UNIMMAP formulation improves pregnancy outcomes and maternal and offspring nutritional status,^5 14^ it has not been demonstrated to fully resolve nutritional deficiencies.^15 16^ Specifically, in a trial of MMS versus IFA with micronutrients in the UNIMMAP formulation^17^ conducted by our group, the prevalence of micronutrient deficiencies in early pregnancy in a subset of trial participants was 2.5% for folate and iodine, 4%–5% for iron, ~35% for vitamin B12 and~60% for vitamins D and E.^16^ At 32 weeks gestation, daily MMS supplementation decreased the prevalence of deficiencies by 15%–40% compared with IFA, but deficiencies of micronutrients such as vitamin B12 and zinc were exacerbated with advancing pregnancy regardless of MMS, suggesting higher need in later gestation. Plasma volume expansion, prioritisation of some micronutrients for the fetus^18^ and other metabolic alterations of pregnancy are known to enhance maternal nutrient requirements during pregnancy. Thus, it is reasonable to hypothesise that more than 1 RDA of a nutrient could be required to best support PW in LMICs.

Importantly, several randomised controlled trials with higher nutritional dosages have shown significant advantages for pregnancy outcomes, some even provided preconceptionally, in both LMIC and high-income contexts.^1^ In Tanzania, twice the RDA for vitamin E and 6–10 times the RDAs for vitamins C and B—but no vitamin A or zinc—reduced low birth weight and small-for-gestational age compared with IFA.^19^ In Ghana, an MMS supplement containing 2× the RDAs of thiamin, riboflavin, niacin, vitamins B6, B12, D and E, zinc, copper and selenium did not impact birth outcomes.^20^ Finally, in Guinea-Bissau, an MMS trial with 2× the RDA of 15 micronutrients increased birth weight by 95 g compared with 53 g for a single RDA supplement versus IFA.^21^ Biomarkers of micronutrient status were not, however, assessed in any of those trials. A variety of single- or dual-nutrient trials at higher than 1 RDA levels have been conducted, including trials using vitamins B12, C, D and E, zinc and selenium, typically in relation to birth outcomes rather than status.^22^,^26^

With this background and knowledge gap, we have embarked on conducting two parallel bioefficacy trials to ascertain the amounts of several micronutrients that will best meet the nutritional requirements of PW and WRA in rural Bangladesh as assessed by biochemical indicators of status over the course of the supplementation. Collectively, the Micronutrient Dose Response (MiNDR) trials will test whether micronutrient intake levels beyond 1 RDA/AIs result in nutrient sufficiency (and without resulting in excess) in women (both non-pregnant/non-lactating and pregnant) residing in an undernourished setting because (1) current recommendations for nutrient intakes might not be appropriate for LMICs, and data derived in women and during pregnancy are limited, (2) single RDAs are unlikely to eliminate most deficiencies during pregnancy, based on our previous findings and (3) some studies that have supplemented with more than 1 RDA have found greater benefits.^23^,^27^ Multiple levels of a wide variety of nutrients are provided in these ongoing trials to model the dose response and identify the lowest amount of each nutrient that minimises deficiency without compromising safety. Secondary objectives are to discover novel functional biomarkers of nutrient status and examine responses based on baseline characteristics and underlying nutritional status.

## Methods and analyses

### Trial setting and design

The MiNDR trial is being carried out at the JiVitA Project site in rural Gaibandha district, Rangpur Division, of northwestern Bangladesh.^27^ This agrarian site occupies a contiguous rural area of 260 km^2^ and comprises a total population of WRA of approximately 60 000. The MiNDR study area was selected to include two Unions (the smallest rural administrative and local government unit in Bangladesh) of the district and has been divided into 75 community clusters called sectors, with the number of households ranging from 150 to 200 each, and supported by one project-hired community health research worker (CHRW) per sector.

**Figure 1:**
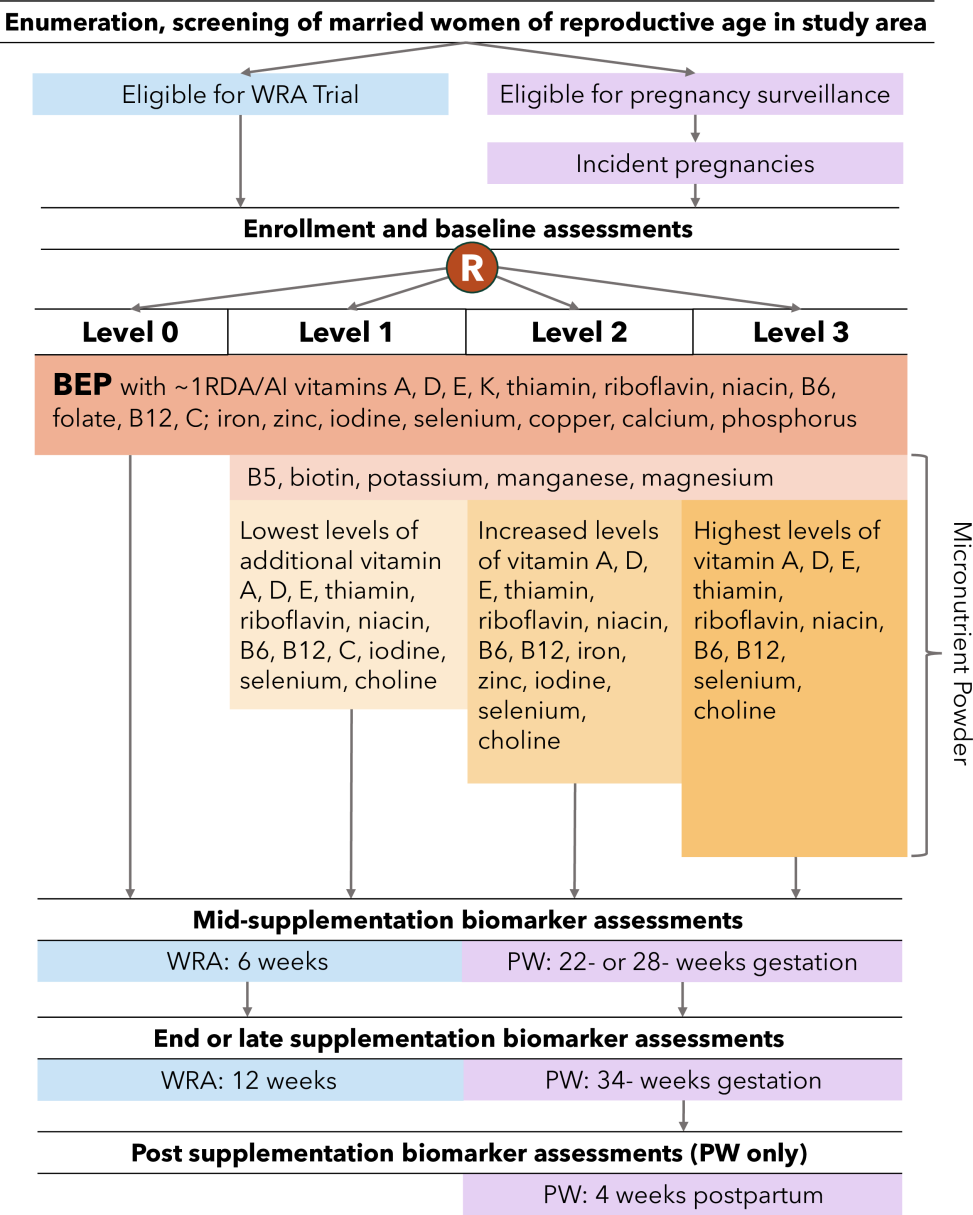
MiNDR Trial study design. BEP, balanced energy protein; MiNDR, Micronutrient Dose Response; PW, pregnant women; R, individual randomisation; WRA, women of reproductive age.

Two parallel dose-response studies each using a four-arm, double-blind, individually-randomised, controlled design are currently ongoing in this study area with the objective to compare three nutrient supplement dose levels between ~1 RDA/AI and below the UL (figure 1). A total of 19 vitamins and minerals are delivered both in the form of a supplement drink at three levels, or a placebo and a daily lipid-based balanced energy and protein (BEP) product containing ~1 RDA/AI amounts of 18 micronutrients including in the control arm. The primary outcomes of interest are biochemical micronutrient biomarkers for the nutrients being tested assessed pre-, mid- and end of supplementation. The trial protocol is reported following the Standard Protocol Items: Recommendations for Intervention Trials checklist (online supplemental file 1).

### Participant recruitment and eligibility

Prior to the start of the trials, a study-wide household census was conducted in October 2023 to identify all married women 18–35 years of age living in the study area and to determine their eligibility for pregnancy surveillance or for the WRA trial in which we sought non-pregnant, non-lactating women not planning a pregnancy for a 6–12 month period. Trained female CHRWs, hired by the project and with a high-school degree or more, went house-to-house within their assigned sectors to identify and meet all the WRAs to administer consent for enumeration and screening.

### Eligibility/ineligibility criteria

Women not currently living with their husbands, pregnant or breastfeeding an infant <12 months were considered ineligible for either trial. Women not currently breastfeeding, not planning a pregnancy and using long-acting family planning methods (hormonal and non-hormonal IUDs, combined injectable contraceptives, tubal ligation or hysterectomy) were considered eligible for the WRA trial.

Any women planning a pregnancy, not using any family planning methods or using ‘temporary’ family planning methods (hormonal birth control pills, condoms, menstruation tracking, others) were considered eligible for an active pregnancy surveillance that was implemented.

Women eligible for pregnancy surveillance are visited every 5 weeks by the sector CHRW to ascertain their last menstrual period (LMP) and provide a urine-based pregnancy test if they have no history of menstruation in the past 30 days. Those with a positive pregnancy test and <16 weeks of gestation according to their reported LMP are immediately scheduled for an enrolment visit, at which time consent for participation in the trial is sought.

In both trials, only consenting women are eligible for enrolment. PW reporting an early miscarriage or an abortion prior to enrolment are not enrolled in the PW trial.

### Study interventions and blinding

#### BEP supplement

The BEP supplement fortified with 1 RDA/AI of a variety of micronutrients and Ca and P, at amounts below the RDA (table 1), is provided to women in all four arms on a daily basis and serves as the vehicle for delivering micronutrients in the control arm. The fortified BEP is a ready-to-eat lipid-based food product made with rice, lentil or chickpea flour, skim milk powder, oil and added sugar, and it provides 382 kcal energy and 14.3 g of protein in each 75 g serving packet. The BEP product addresses an energy and protein gap related to the increased requirements during pregnancy and was considered appropriate to provide to all women in the four arms. The BEP product is manufactured locally by Care Nutrition, Dhaka, Bangladesh.

**Table 1:** Micronutrient content of BEP and test products by intervention arms*

Nutrient		Total MN by level+BEP††			RDA/AI†		UL‡
	BEP+level 0	BEP+level 1	BEP+level 2	BEP+level 3	WRA	PW	
Vitamin A (IU)	2564	3746	5661	7492	2333	2567	10 000
Vitamin D (IU)	600	1400	2200	3000	600	600	4000
Vitamin E (IU)§	24	75	186	298	33.3	33.3	1000
Vitamin B1 (mg)	1.4	2.8	4.2	5.6	1.1	1.4	ND
Vitamin B2 (mg)	1.4	2.8	4.2	5.6	1.1	1.4	ND
Vitamin B3 (mg)¶	18	35	65	100	14	18	ND
Vitamin B6 (mg)	1.9	4	6	10	1.3	1.9	100
Vitamin B9 (µg)	400	400	400	400	400	600	1000
Vitamin B12 (µg)	2.6	6	12	18	2.4	2.6	ND
Vitamin C (mg)	100	120	120	120	75	85	2000
Iron (mg)	30	30	40	40	18	27	45
Zinc (mg)	15	15	20	20	8	11	40
Iodine (µg)	220	220	290	290	150	220	1100
Selenium (µg)	65	100	200	300	55	60	400
Copper (mg)	1	1	1	1	0.9	1	10
Choline (mg)	--	550	750	900	425†	450†	3500
Calcium (mg)	500	500	500	500	1000	1000	2500
Phosphorus (mg)	383	383	383	383	700	700	4000/3500
Vitamin K (µg)	90	90	90	90	90†	90†	ND
Vitamin B5 (mg)	--	7	7	7	5†	6†	ND
Biotin (µg)	--	35	35	35	30†	30†	ND
Potassium (mg)	--	1000	1000	1000	2600†	2900†	ND
Manganese (mg)	--	2.6	2.6	2.6	1.8†	2†	11
Magnesium (mg)**	--	145	145	145	310	350	350

* Intervention arms will receive BEP+MN powder with any of the three levels (1–3) of micronutrient powders, and control arm will receive BEP+placebo powder (level 0) with the same properties but no nutrients.

† Dietary reference intakes are reported as AI (†) when RDA are not available.^6^,^10^

‡ The ULs for all nutrients are the same between WRA and PW, except for phosphorus (WRA-4000 mg, PW-3500 mg).

§ RDA and UL are reported as synthetic forms of α-tocopherol.

¶ Vitamin B3 is provided as niacinamide in the test products. UL for vitamin B3 is based on the levels of nicotinic acid associated with skin flushing, and nicotinamide does not cause skin flushing.

** RDA considers magnesium from all dietary sources, while UL includes magnesium only from dietary supplements and medications.

†† BEP product contains protein—14.3 g, fat—21.8 g, carbohydrate—32.2 g (including 9.6 g of sugar) and energy—382.4 kcal per serving (75 g).

AI, adequate intake; BEP, balanced energy protein; MN, micronutrient; ND, not determined; PW, pregnant woman; RDA, recommended dietary allowance; UL, tolerable upper intake level; WRA, women in reproductive age.

#### Micronutrient (MN) powder supplement

In each of the study arms, one of three varying doses of micronutrients or a placebo is provided daily in a powdered form in a sachet and reconstituted as a drink with 100 mL water (table 1). The drink has a mango-orange flavour that was tested for cultural acceptability prior to the study. The micronutrient (MN) powders were manufactured by DSM-Firmenich in Basel, Switzerland, specifically for this trial and were shipped to Bangladesh following verification of the micronutrient contents, stability and other quality assurance testing. The MN powder sachets are identical in weight, shape, size and colour and were labelled with codes A, B, C and D by the manufacturer, who generated the allocation codes. The codes are kept in a sealed envelope at a central location at DSM-Firmenich and by icddr,b staff not involved in the study to preserve the blinding of the investigative teams.

The formulations for WRA and PW trials are identical, and the nutrient content in each of the four arms is detailed in table 1. The amount of each micronutrient to be included was individually determined based on an extensive review of the literature, inputs from a technical advisory group with expertise in micronutrients and considering data on micronutrient status of women from previous studies in this setting. Typically, nutrient levels from the BEP and powdered supplement combined range from 1 RDA/AI in the control group to up to 75% of the UL, for those nutrients where ULs were determined. Contributions of usual diet and micronutrient overages in supplements were considered in determining the three levels of doses, as were saturation doses of nutrients for nutrients with no defined UL or ULs that are particularly high (vitamins C and E) and known micronutrient interactions (such as iron and zinc). A recent review examining existing single nutrient trials, the amounts of micronutrients provided (sometimes many times the RDA), timing and duration of supplementation during pregnancy and outcomes for mothers and offspring was used to guide the selection of doses.^1^ A recent paper examining the risk of excess micronutrient intakes in pregnancy from antenatal supplements^28^ that documented that only iron and niacin would potentially exceed the UL when considering diets and other sources such as fortification also provided useful guidance. Other practical considerations that informed the design of micronutrient products and were evaluated in a formative phase of the trial were whether micronutrient supplements could be provided as tablets and how to distribute micronutrients between the powdered products or as part of the BEP, given the contribution of some nutrients to bulk and organoleptic properties.

In this bioefficacy dose-response trial, adherence to the supplementation is achieved through direct daily observation of the consumption of the micronutrient drink (or follow-up via phone if not possible), with study-hired CHRWs visiting the participants’ homes, helping with reconstituting the drink, replenishing supplies and counting and retrieving empty BEP and powder packets. At the start of the supplementation, participants are advised not to take any other dietary supplements while in the trial.

### Outcomes

**Figure 2:**
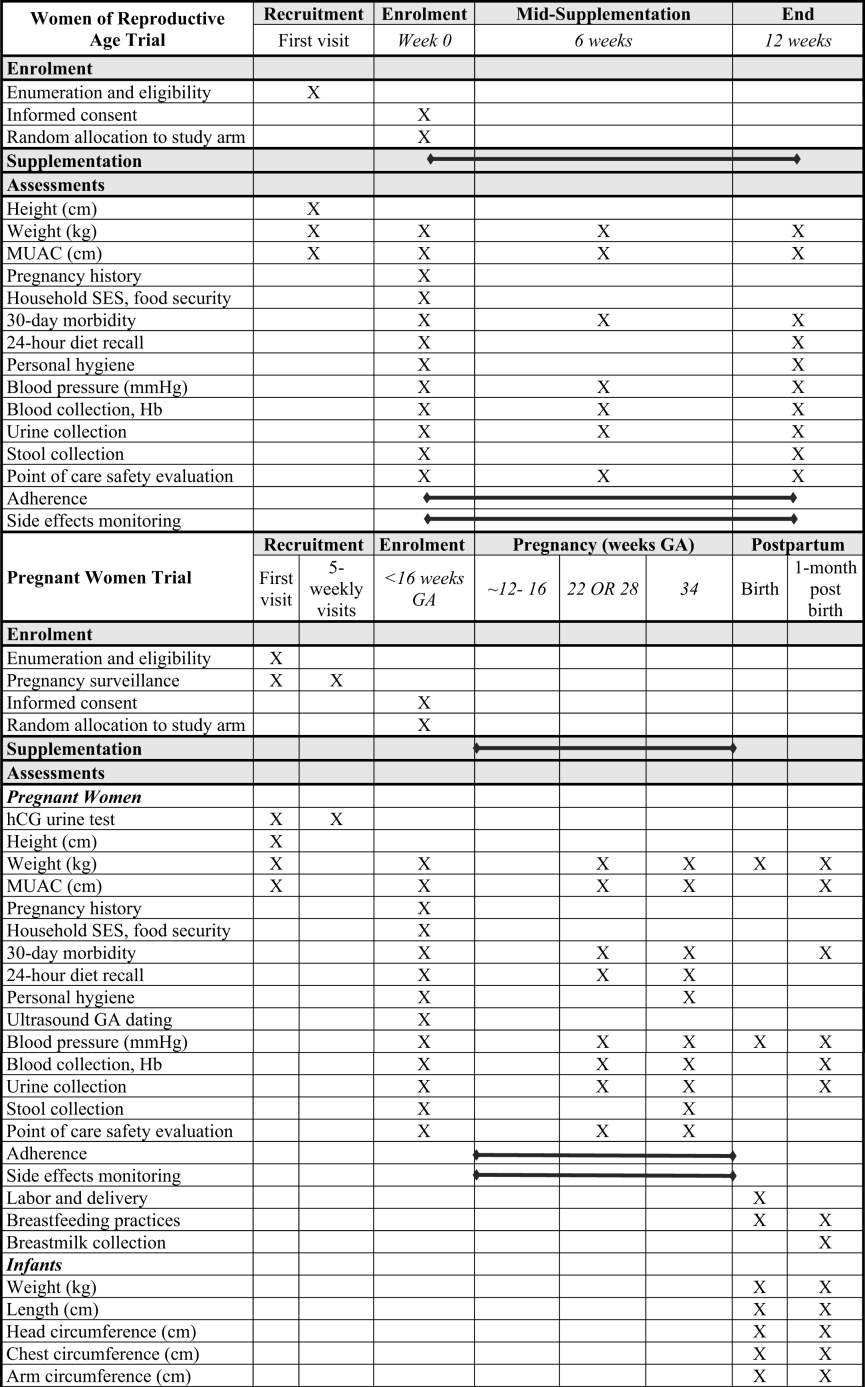
MiNDR trial participant timeline for enrolment, supplementation and assessments. GA, gestational age; Hb, haemoglobin; hCG, human chorionic gonadotropin; MiNDR, Micronutrient Dose Response; MUAC, mid-upper arm circumference; SES, socioeconomic status.

The primary outcomes to evaluate dose response are listed in table 2 and will be assessed in serum or plasma and urine collected at the time points indicated in figure 2. They include micronutrient status biomarkers specific for the micronutrients that are varied in the study arms. Biomarkers were chosen to reflect the continuum of micronutrient status from deficiency to excess, largely summarised by Gibson,^29^ but also derived from searches of the more recent literature. Examining a spectrum of status will allow us to model nutrient amounts required to best support micronutrient sufficiency in women while minimising both deficiency and excess. Biomarkers may be static (ie, the nutrient or a nutrient metabolite in circulation or as an excretory product) or functional (ie, the consequence of nutrient deficiencies or excesses),^29^ and where possible each type was selected to elicit complementary information.

**Table 2:** MiNDR study primary outcomes for nutrients provided at multiple levels in the trial intervention arms^29^

Nutrients	Biomarkers of status
Vitamin A	Plasma retinol, retinyl esters and serum retinol binding protein
Vitamin D	Serum 25-hydroxyvitamin D
Vitamin E	Plasma α and γ tocopherol
Vitamin B1 (thiamin)	Erythrocyte transketolase enzymatic activity, urinary B1 excretion
Vitamin B2 (riboflavin)	Erythrocyte glutathione reductase enzymatic activity, plasma B2 metabolites, urinary B2 excretion
Vitamin B3 (niacin)	Urinary N-methylnicotinamide and 2-pyridone
Vitamin B6 (pyridoxine)	Plasma pyridoxal-5-phosphate, urinary B6 excretion
Vitamin B12	Serum B12, holo-transcobalamin, plasma methylmalonic acid, and homocysteine
Iron	Serum ferritin and soluble transferrin receptor
Iodine	Urinary iodine and plasma thyroglobulin
Selenium	Serum selenium, plasma glutathione peroxidase
Zinc	Serum zinc

MiNDR, Micronutrient Dose Response.

The faecal microbiome will be assessed as a secondary outcome. Other secondary outcomes include assessment of clinical biomarkers for safety evaluation, hormonal, inflammatory and bone turnover biomarkers for the interpretation of primary outcomes and breast milk vitamin and mineral assessments (in the PW trial).

### Sample size

The principal objective of the study is to characterise the dose response and to the extent possible pharmacokinetics of micronutrients with reasonable precision^30^ to allow for estimations of optimal intakes of supplemental nutrients to support adequate but not excess micronutrient status of women. The study was powered to achieve a 95% CI ranging from 60% to 140% of the geometric mean estimates of key pharmacokinetic parameter (ie, clearance) for each nutrient within each subgroup, with at least 80% power. Variability estimates from previously collected data^16^ based on steady-state concentration (Css) were considered as surrogates for variability on clearance (approximately 190% variability was observed for thyroglobulin as a measure of iodine status) to perform simulations to assess sample size and number of samples per subject. Those data were from an antenatal MMS trial of~1 RDA of a variety of the nutrients included in the WRA and PW trials versus iron-folic acid alone, as standard of care, conducted at the same study site.^16^ To balance participant burden and sampling requirements, 2–3 sample time points post-baseline were selected for WRA and PW. Using these sampling measures, the sample size of about 60 participants per dose group was estimated to achieve the set precision criteria.^30^ With an anticipated loss to follow-up ranging from 10%–20% (higher in pregnancy due to early fetal loss), we plan to enrol up to 350 pregnancies in the PW trial and 300 participants in the WRA trial to achieve final sample sizes of 240 WRA and 240 PW per trial, evenly distributed across intervention groups.

### Sampling and randomisation

For the WRA trial, all eligible WRAs identified through census and enumeration activities were stratified into three age groups (18–23 years, 24–28 years and 29–35 years) and ordered by geographic area and household number to serve as a sampling frame. 100 eligible WRAs were randomly sampled from each age stratum to obtain a target sample size of 300. A randomisation sequence was pregenerated by the study statistician in Bangladesh using randomly permuted blocks of size 4 to ensure similar sample sizes across the four study groups. Prior to the study start, the randomly selected WRAs were allocated sequentially to a study group using the allocation sequence list for each age group. If a WRA refuses participation in the trial or is not met after consecutive home visit for enrolment, another eligible WRA in the same age stratum is randomly selected from the sampling frame, consented and assigned this allocation code at the time of enrolment.

For the PW trial, a randomisation sequence was pregenerated using randomly permuted blocks of size 4. As women in the study area are identified as pregnant by the study’s pregnancy surveillance system, they are sequentially allocated to a study group following the randomisation sequence list. If a PW refuses participation or is not met after consecutive visits after pregnancy detection, she is not enrolled, and the next incident PW is assigned this allocation code.

The allocation code for each individual study participant is revealed to a senior female interviewer (FI) at the time of the enrolment visit once it is completed on the digital tablet that is used for data collection by connecting to the central study database. The randomisation procedures were performed in Stata 17.0 (StataCorp, College Station, Texas, USA).

### Trial methodology

Figure 2 details the trial procedures, including assessments at each study visit for both trials. Data collection activities are similar across the WRA and PW trials, although the duration of supplementation and number of visits differ between the WRA and PW trials. The schedule for visits is driven by when the primary outcomes of status, requiring blood drawing, are assessed. For WRA, the supplementation duration and follow-up are 3 months (12 weeks), with baseline (presupplementation), a mid-trial (6 weeks) and end of supplementation assessment and biospecimen collection. This duration was selected based on expert consensus and experience in dosing trials among our technical advisory group. In the pregnancy trial, gestational age dating is performed by ultrasound following pregnancy detection, after which all women undergo a baseline assessment. For the midline assessment, women are systematically (alternatively) assigned to either a mid-pregnancy visit at either 22 or 28 weeks of gestation, with all women assessed in late pregnancy (~34 weeks gestational age). Supplementation continues until birth, and PW are visited in their homes or in the facility after delivery to assess birth anthropometry and other conditions in the mother and the newborn. A final visit at 1 month postpartum allows for the additional collection of a human milk sample. The duration of supplementation was chosen to mimic the time that women would be advised to take a pregnancy supplement and to be able to characterise the effects of gestational age on biomarker concentrations.

#### Data collection

Different cadres of trained project-hired staff conduct home visits for data collection requiring specific assessments listed in figure 2. CHRWs (n=45) conduct pregnancy surveillance and supplementation visits and record observed daily supplement consumption; FIs (n=5) are trained data collectors specialised in questionnaire-based interviews and newborn anthropometry; study technicians (STs, n=4) are trained in phlebotomy, anthropometry and other clinical techniques; biospecimen collectors (BSCs, n=3) are dedicated to collecting urine and faeces, which require special timing and cold chains and trained lab technicians (LTs, n=2) process biospecimens using standard operating procedures. Ultrasound technicians (UTs, n=2) are rigorously trained in ultrasound-based gestational age dating and certified by a radiologist to perform ultrasonography. All activities are conducted with day-to-day management by a local team of experts in technical and administrative aspects of trial conduct, including research physicians who supervise ultrasound and other assessments.

Enrolment in either trial consists of written informed consent and baseline assessments spread out over several days. In the PW trial, ultrasound-based gestational dating is done in participant homes by the UT with portable instrumentation (Mindray Z60) using the INTERGROWTH-21st protocols for early pregnancy.^31^ At the baseline assessments in both trials, FIs do an interview to collect data on pregnancy history, a 30-day morbidity history, household socioeconomic status and a 24-hour dietary intake recall. For the 24-hour recall, FIs use the multiple pass method, along with a locally produced food list and photo atlas to optimise recall of the types and amounts of foods consumed in the previous 24 hours. To assess the usual variance of certain nutrients in the population, a replicate visit in the same week but on a non-consecutive day is conducted at baseline for one-third of the enrolled women in the WRA trial using systematic sampling.

On the day following enrolment in the study, the ST visits the participant’s home to collect blood using venipuncture and measures height, weight, mid-upper arm circumference (MUAC), haemoglobin using venous blood (Hemocue 301, Hemocue, Sweden) and blood pressure using the Microlife (B3 Basic) digital blood pressure machine. Finally, the BSCs collect faeces and the first urine output of the day on the following day. Most assessments and biospecimen collections are repeated at the subsequent visits, although some interviews are only done at baseline and faeces are only collected at baseline and at the end of the supplementation period (figure 2).

Once all baseline assessments have been conducted and biospecimens collected, the CHRW visits the woman in their home to begin supplementation generally between 12 and 16 weeks of gestation in the PW trial or immediately for those in the WRA trial. The CHRW prepares the drink and directly observes the participant consuming it, although the BEP supplement is allowed to be finished later.

A birth notification system set up to alert the project staff (generally via a phone call) that results in FIs reaching women and their newborns within 72 hours of birth to measure newborn and maternal anthropometry and collect information on labour and delivery. A final visit is conducted at 1-month postpartum to collect postpartum blood, urine and breast milk specimens along with data on infant vital status, infant and maternal anthropometry, breastfeeding practices, maternal postpartum morbidity and 24-hour dietary recall at this visit. In cases where a woman moves out of the study area, every attempt is made to meet the woman within a reachable distance to provide supplements and conduct the study visits. Women are considered lost to follow-up if they move out of the study area permanently or refuse to continue participation in all study activities. Reasons for withdrawal are recorded.

#### Biospecimen collection, processing, storage and shipment

All biological samples (blood, urine, faeces and breast milk samples) are collected by trained study technicians during home visits according to standard operating procedures. A total of 12 mL of venous blood is collected from each participant in two evacuated tubes, a lithium heparin tube and a serum trace element free tube (Becton Dickinson, USA). Blood samples are collected in the morning, ideally in a fasted state, to minimise the contribution of diurnal variability to the imprecision in biomarker assays and to ensure that biomarkers do not reflect postprandial conditions. Women also self-collect 20 mL midstream urine samples from the first void of the day in 70 mL sterile collection cups (STARPLEX, Cleveland, Tennessee, USA), and faeces samples (~1 g or 1 mL in volume) in DNA/RNA shield faecal collection tubes (Zymo Research Corporation, USA) to preserve both DNA and RNA for metagenomics and transcriptomics, respectively. Breast milk sample collection in the PW trial entails a full breast expression using an electric breast milk pump (Medela Symphony, USA) at 1-month postpartum. A milk sample of 10–15 mL is transferred into sterile trace element-free containers (Fisher Scientific, USA) to be used for biomarker analysis. Biospecimens are protected from light to ensure stability of the photosensitive vitamins and transported from the households to the field laboratory in a cold chain for processing and storage within a few hours of collection in the field.

Whole blood samples are centrifuged at 3000×g for 15 min at 4°C to obtain aliquots of plasma from the lithium heparin tubes and serum from the trace element-free tube. Prior to centrifugation, an aliquot of whole blood is also obtained from the lithium heparin tube. The lithium heparin tubes are further processed to obtain a haematocrit measure, red blood cell (RBC) pellets and buffy coat aliquots (on QIAcard FTA cards, Whatman). The whole blood, plasma, serum, RBC pellets and urine samples are aliquoted into sterile and prelabelled cryovials and stored in a −80°C freezer. Buffy coat cards are stored at room temperature. The DNA/RNA shield faecal collection tubes are directly stored at −20°C and later shipped to the icddr,b Genome Centre (iGC) for metagenomic sequencing. A creamatocrit measure is obtained from breast milk, and aliquots of breast milk are stored at −80°C. For micronutrient biomarker analyses, samples are shipped to the Immunobiology, Nutrition and Toxicology laboratory at icddr,b (Dhaka, Bangladesh) or the Programme in Human Nutrition Laboratory at Johns Hopkins Bloomberg School of Public Health (Baltimore, USA) using dry ice, liquid nitrogen or ice packs, depending on the sample type. Once they are received, they are stored again in −80°C freezers until thawed for analysis.

#### Safety evaluation and side effects monitoring

Nutrients are being provided in amounts that do not reach the UL, such that the micronutrient doses provided are considered safe for daily consumption. Nonetheless, participant safety is rigorously monitored in the trials. A study clinician is designated as a safety monitor. Safety evaluations are conducted through three different standard procedures created specifically for this study. The first line of assessments entails the use of clinical markers tested using a point-of-care system to identify women who face a potential risk and are discontinued from the trial prior to supplementation. The second tier of assessments involves weekly monitoring of side effects that could be associated specifically with consumption of high levels of individual nutrients (table 3). They were selected based on the possibility of being linked to excess nutrient intakes,^6^,^10^ although they are largely non-specific. The third line of safety monitoring involves timely reporting of adverse events (AEs) to the Principal Investigator (PI) and data safety and monitoring board (DSMB). Details of these procedures are described below.

**Table 3:** Symptoms for weekly evaluation of side effects and considerations of severity^6^,^1028^

Symptom	Rationale	Determination of severity
Nausea and/or vomiting	Can be associated with excess nutrients including vitamin A, vitamin D, vitamin E, niacin	Severity based on number of episodes per week and per day
Gastrointestinal distress: diarrhoea and/or constipation	Can be associated with excess iron, zinc, selenium	Based on frequency per week and per day, evidence of dehydration and limitation of usual activities
Weakness and/or fatigue	Can be associated with excess vitamin E	Degree to which daily activities are affected
Headache	Can be associated with excess vitamin A, vitamin E, zinc	Degree to which daily activities are affected
Hair and/or nail brittleness	Can be associated with excess selenium	Based on history and clinical judgement
Rashes	Can be associated with excess selenium; flushing associated with excess niacin	Extent of appearance
Fishy odour	Associated with excess choline	Based on history and clinical judgement—perception of newness relative to supplements
Excess or irregular bleeding (nasal, oral, ocular)	Can be associated with excess vitamin E or insufficient vitamin K	Always considered severe
Black tarry stool	Can be indicative of blood loss (vitamins E and K)	Always considered severe
Bruising without injury	Can be associated with excess vitamin E or insufficient vitamin K	Always considered severe
Blurred vision	Can be associated with excess vitamin A	Always considered severe
Tingling of fingers and/or toes	Can be associated with excess vitamin B6	Always considered severe

Clinical biomarkers are assessed using a point-of-care instrument (Piccolo Xpress, Abbott, USA) at enrolment prior to starting supplementation and during the follow-up visits to identify potential hepatic or renal conditions that could affect the participant’s ability to metabolise nutrients. Evidence of liver or kidney dysfunction, in addition to reported symptoms if any, is evaluated by a team of research physicians and the safety officer who determine whether a participant should be disenrolled (at baseline). If these conditions develop or are identified at the follow-up visits, the RPs determine if the participant is experiencing adverse effects due to the study supplements and/or needs to be referred for care for reasons unrelated to supplementation. At the midline visits, if the RP determines that the supplement may be the cause of the elevated levels, the woman is discontinued from the study and supplementation and additional visits, with the exception of weekly morbidity assessments that continue until the end of the planned follow-up period. At the late pregnancy visit, any evidence of elevated clinical markers identifying risk results in the participant being asked to stop the MN and BEP supplements, although they would not be discontinued from the study for the birth and postpartum visit. Reference cut-offs that trigger potential discontinuation and/or referral following other clinical assessment are listed in online supplemental file 2 and include elevated liver enzymes, electrolytes and evidence of renal dysfunction. Other clinical indicators include plasma glucose, phosphorus, chloride and a lipid panel (Piccolo Xpress), which will be characterised by study arm at the end of the trials and are also used by the RPs as ancillary measures, along with blood pressure, as part of their safety review. Women identified with severe anaemia (haemoglobin <7 g/L) are not enrolled in the study and are provided referral for treatment; women with incident cases of severe anaemia at follow-up visits are referred for care but retained in the study.

Weekly side-effect reports from the participant ascertained by CHRWs are reviewed by two study RPs assisted by a trained nurse. A phone call is made to those reporting symptoms to elicit more details, and based on their report and RPs’ clinical judgement, the condition is assigned a level of severity (mild, moderate, severe) using clinical criteria (table 3). All participants with moderate and severe symptoms are visited in the home by a study RP. At the visit, following further clinical examination, the RPs decide whether to (1) provide counselling and reassurance that symptoms are transient and/or unrelated to the supplements, (2) provide such counselling but also provide a referral for care, (3) provide a referral for care without pausing supplementation while scheduling another visit for 7 days later to ensure no exacerbation or resolution of symptoms or (4) provide a referral, pause supplementation, and schedule another visit after 7 days (online supplemental figure 1). After further follow-up visits, the RP may recommend continuing or permanently stopping supplementation. In the case of a referral, transportation for the participant and family member accompanying them is provided to and from the health facility.

Serious AEs (SAEs) include maternal hospitalisation, life-threatening conditions, infant gross congenital anomalies and maternal or infant death. Knowledge of the event is reported to the study principal investigator (PI), who submits an initial report to the ethical review committee (ERC) of the icddr,b and the DSMB chair within 24 hours of the event. Further reports are provided to the PI, the ERC and the DSMB chair until the SAE is resolved. All AEs are investigated by study physicians and reviewed with the safety monitor, who finalises and prepares the reports to the PI. All AEs and SAEs are reported annually to the Johns Hopkins Bloomberg School of Public Health (BSPH) IRB. Only unanticipated events are reported immediately to the BSPH IRB (in addition to the icddr,b ERC).

### Laboratory analyses

Primary outcomes (table 2), ancillary biomarkers that aid in their interpretation (eg, biomarkers of inflammation), and other non-specific biomarkers potentially responsive to nutrient intake will be assessed at the JiVitA project laboratory in Rangpur, at icddr,b, in Dhaka or in the micronutrient research laboratory of the Center for Human Nutrition at BSPH in Baltimore.

Point-of-care tests for liver and kidney function, selected minerals and electrolytes, plasma glucose and a lipid panel (Piccolo Xpress, Abbott, USA), a haematocrit measurement and creamatocrit measurement in milk are performed at the time of biospecimen collection in the field laboratory at JiVitA.

At icddr,b, serum-based assays including ferritin and soluble transferrin receptor, circulatory total B12, 25-hydroxy vitamin D, folate and α−1 glycoprotein (inflammation) will be analysed using automated clinical chemistry analysers (Cobas c311 and Cobas e601, Roche Diagnostics GmbH, Germany). Serum retinol binding protein (enzymatic immunoassay) and urinary iodine (Sandell–Kolthoff reaction) will be analysed using conventional 96-well plate methods.

At the Center for Human Nutrition, vitamin A (retinol and retinyl esters), E (α- and γ-tocopherol), and B vitamers (for thiamin, riboflavin, niacin and vitamin B6) will be analysed by ultra-performance liquid chromatograph methods (UPLC-photodiode array detector and UPLC-fluroscence detector, Waters Acquity, USA) in plasma, breast milk and urine (specifically B vitamin excretion products). Plasma erythropoietin (iron), thyroglobulin (iodine), parathyroid hormone (vitamin D and calcium), C-reactive protein (inflammation), whole blood folate, urinary pyrilinks-D (bone turnover, vitamin A, D and calcium) will be measured by chemiluminescent immunoassay (IMMULITE 2000, Siemens, USA). Functional assays of plasma vitamin B12 (holotranscobalamin and methylmalonic acid), hormonal assay of iron (hepcidin) and functional enzymatic assays for thiamin, riboflavin and selenium will be analysed by 96-well plate methods. Mineral panels (copper, selenium, zinc, iron, manganese, magnesium, calcium, potassium and phosphorus) in serum and breast milk will be analysed by inductively coupled plasma mass spectrometry (7850 ICP-MS; Agilent, USA).

Additionally, faeces will be assessed for the faecal microbiome profile using 16S ribosomal RNA sequencing. Rationale and more detailed methods for selected biomarkers will be provided in a separate publication.

#### Data management

Data collection activities are scheduled centrally using a web-based scheduling system developed by the Data Management Centre daily according to individual participant timelines. Completion of data collection forms is monitored daily or weekly as appropriate (ie, daily supplementation vs weekly symptom collection vs periodic visits). All data collection cadres use password-protected Android tablets and Enketo (web browser-based, https://enketo.org/) forms.^32^ Data submissions are initially stored on Ona^33^ data servers, which are encrypted to industry standards and are transferred via application programming interface (API) to the study’s database on an encrypted server. Ona is a third party organisation that works with humanitarian and other organisations to improve how they collect data.^34^ Data collection forms are designed and tested through the Enketo forms and submitted to the Ona data server. Entry restrictions (data range, plausible values) are applied to reduce data entry errors. Biospecimen throughput is managed through a ‘labtrack’ system, where at each step of collection, processing, storage and transportation, the biospecimen barcodes are scanned and linked to participant records and/or study management information. Data is uploaded and stored on an encrypted server on a weekly basis and undergoes regular data quality checks. Missing or improbable data are investigated by field staff and quality control monitors to investigate any unusual values, patterns and entry errors in the datasets.

Digital data security is constantly monitored and upgraded to reflect state-of-the-art guidance for human subjects’ data protection. Digital devices in the field are all password-protected and managed using a device fleet management system. This system allows administrators to monitor, at the individual device level, when devices are turned on or off, levels of charge, and alerts administrators if devices are tampered with (eg, attempted change in subscriber identity module). Devices can be locked and wiped remotely, thwarting any efforts to steal or tamper with data. All data transmissions are conducted in an encrypted fashion, using Health Insurance Portability and Accountability Act (HIPAA) grade cloud storage systems. Data are backed up continuously to US-based commercial cloud storage solutions (Dropbox Business Account). Dropbox is designed with multiple layers of protection, including secure data transfer, encryption, network configuration and application-level controls distributed across a scalable, secure infrastructure.

Analytical datasets will be deidentified and stored on an encrypted server on an internal network behind a firewall. Access to identifiable data is restricted to the PI, key coinvestigators and senior staff. Only deidentified datasets will be shared beyond the study team.

### Data analysis

The main analysis is based on dose response to supplementation by assessing biochemical indicators of nutrient status in the plasma and urine samples as described in table 2. Data analysis will be conducted separately for each trial. The main outcomes include nutrient status biomarkers and nutrient-specific biomarkers of excess or non-specific clinical or adverse side effects assessed over time. The minimum dose and time at which sufficiency is achieved (ie, with no indication of excess) will be estimated for each nutrient. The primary analysis uses a pharmacokinetic approach to estimate dose response. The analysis population will include subjects having a baseline and at least one post-baseline measurement. Secondary objectives of the study are to discover novel functional biomarkers of nutrient status, examine response based on baseline status and underlying nutritional status and genomic and microbiome characteristics in addition to other baseline factors collected in the study.

The Css data collected from the study will be analysed using one of two approaches: where previous literature is available that specifies a relevant population pharmacokinetic model;^35 36^ the objective will be to estimate the pharmacokinetic (clearance and volume of distribution) parameters of the given nutrient/biomarker. In cases where literature is not available, we will employ linear or non-linear mixed-effect modelling to account for individual baseline variations in micronutrient status. Both of these approaches will include the four following steps: (1) a base model for each nutrient developed, motivated by previously published reports where available; (2) a full covariate (prognostic-factor) search conducted to correlate the individual pharmacokinetic parameters with subject-demographic and baseline parameters; (3) the final model will be qualified using quantitative predictive checks, standard goodness of fit plots, and individual predictions of the concentration-time profile to evaluate model performance, and model-based estimates and bootstrap methods, providing 95% CIs where appropriate will be used to evaluate parameter robustness and precision and (4) simulations performed to inform the appropriate doses of micronutrients to meet the selected target concentration. The selected target concentration will be informed by the literature, status biomarkers, markers of excess and clinical expertise. All steps in the model development process will be documented.

To determine whether the intervention effect on micronutrient concentration is modified by microbiota composition, multivariable linear regression models will be fitted with micronutrient status as the outcome, including an interaction term for (1) treatment-by community state types or (2) treatment-by principal coordinate axis for each of the first two principal coordinate axes. A separate model will be fitted for each micronutrient of interest. Each model will also include covariates chosen a priori that are predictive of micronutrient status to improve the precision of treatment effect estimates. P values will be adjusted for multiple hypothesis testing to preserve the false discovery rate.^37^

### Study oversight and management

The study is being provided overall scientific, ethical and other oversight by an executive team led by the PI and study investigators who meet on a weekly basis to discuss all aspects of the implementation of the trial. The investigative team comprises faculty from BSPH, icddr,b and senior team members of JiVitA. The study protocol and details of forms, procedures and manual of operations, also used to train the data collection teams, were designed and finalised by the executive team. Data collection forms and procedures are translated into the local Bangla language. The cadre of field staff responsible for data collection is supervised by a field supervisor supported by a quality control officer. The field-based, icddr,b and Center for Human Nutrition laboratories are provided oversight by senior investigators under whose supervision the laboratory analyses will be carried out. The data management centre in JiVitA is led by a senior data manager, two programmers and an IT officer and supported by a quality control officer and study statistician. They are in turn supported by a data specialist and statistician based at BSPH. Data analysis in the study will be conducted in collaboration with pharmacokinetic experts at the University of Maryland. Weekly field visits by senior team members in JiVitA and extensive and frequent visits by the PI and study investigators are key for the smooth operations of the project.

### Patient and public involvement

Participants were not involved in the planning of this study. The study was planned and is being implemented jointly by personnel at the Johns Hopkins Bloomberg School of Public Health, Baltimore, Maryland, USA; The JiVitA Project, Rangpur, Bangladesh; and the International Centre for Diarrhoeal Disease Research, Bangladesh (icddr,b), Dhaka, Bangladesh. A workshop will be planned at the completion of the study to communicate the study’s findings to the community at large.

## Ethics and dissemination

###  Ethical approvals

Ethical approvals were obtained from the research review committee (RRC) and ethical review committee (ERC) of icddr,b (protocol no: PR-22078; V.1.3; 7 September 2023). The study has been approved by the Bloomberg School of Public Health IRB at Johns Hopkins University in Baltimore, Maryland. Important protocol modifications are communicated to the IRBs and reported on ClinicalTrials.gov. Consent procedures adhere to the precepts of the Declaration of Helsinki and the Belmont Report. Oral consent is obtained by experienced field workers for the enumeration and pregnancy surveillance activities. Written informed consent, including an agreement to study participation, data sharing and storage of biospecimens for future analysis, is obtained by a cadre of trained FIs for enrolment into the trial, and a copy of the consent document is provided to participants. All study amendments are reviewed by both IRBs and approved. Continuing reviews and annual approvals are also done by both the JHU and icddr,b IRBs. Model-informed consent forms for both trials are included as online supplemental file 3 and Online supplemental file 4.

### Data safety and monitoring board (DSMB)

The ERC at icddr,b is responsible for forming a DSMB, which was established in the preparatory phase of the research to review the protocol and set up terms of reference. The DSMB is charged with providing guidance on the safety and efficacy of the interventions. Each trial will be examined separately for these aspects. An initial prestudy meeting was held on 30 March 2023, with subsequent review of trial progress held on 23 April and 18 December, 2024. The DSMB will meet again midway and at the end of each trial. Interim analysis to examine dose response will be limited since lab-based outcomes of nutrient status will only be available after the intervention periods have ended. The DSBM is responsible for advising the unblinding of the supplementation codes during the trial in case of significant safety concerns. The data, results and other findings resulting from this study will be presented to the DSMB once all the data are available.

### Availability of data and materials

Data will be available on reasonable request to PI, following guidelines of the Johns Hopkins University and icddr,b Institutional Review Boards and local regulations. Following completion of the study, we anticipate taking up to 2 years to publish primary and secondary outcome papers. Data will be provided to the funder and other groups when requested after at least 2 years of completion of the grant period and using data-sharing agreements. Participant-level data when shared will be deidentified. The full protocol, data collection forms and statistical codes can be provided on request.

### Dissemination

Following the breaking of the intervention codes, the data will be analysed, and results of the study will be published within a year of completion of the laboratory analysis. The International Committee of Medical Journal Editors guidelines will be used to establish authorship on papers. Investigators who contributed to the design and implementation of the study and interpretation of the study findings will be included as authors. Study participants and field staff will be acknowledged.

### Trial status

Enrollment for the PW trial and WRA trial began on October 22, 2023, and February 11, 2024, respectively, and concluded in July and September 2024, respectively, followed by an additional 3-7-months follow-up. As of November 30, 2024, 354 pregnant women and 289 WRA have been enrolled.

## Supplementary material

10.1136/bmjopen-2024-090108 online supplemental file 1

10.1136/bmjopen-2024-090108 online supplemental file 2

10.1136/bmjopen-2024-090108 online supplemental file 3

10.1136/bmjopen-2024-090108 online supplemental file 4
