# Supplementary material for: Micronutrient dose response (MiNDR) study among women of reproductive age and pregnant women in rural Bangladesh: study protocol for double-blind, randomised, controlled trials
Source: BMJ Open. 2025 Jan 4;15(1):e090108. doi: 10.1136/bmjopen-2024-090108 (PMC11749533; doi:10.1136/bmjopen-2024-090108)

Supplementary Table 1. Clinical indicators and cut-offs used for participant discontinuation in the MiNDR study

| **Analytes** | **Reference cutoffs** | **Indication of clinical condition** |
| --- | --- | --- |
| Hemoglobin (g/L)^1^ | <7 | Severe anemia |
| Alanine transaminase (IU/L)^2^ | > 60 | Non-alcoholic fatty liver disease |
| Aspartate transaminase (IU/L)^2^ | > 46 |  |
| Calcium (mg/dl)^3^ | <8.8 | Hypocalcemia |
|  | >10.4 | Hypercalcemia, potential Ca/vitamin D toxicity |
| Sodium (mmol/L)^4^ | <136 | Hyponatremia; could be associated with vomiting/diarrhea or heart, renal, liver disease |
|  | >145 | Hypernatremia; could be associated with dehydration or kidney dysfunction |
| Potassium(mmol/L)^4^ | <3.6 | Hypokalemia: can be due to kidney or heart disease—OR vomiting/diarrhea and could benefit from supplements; values <3.0 require immediate referral |
|  | >5.0 | Hyperkalemia; should not be provided with potassium-containing products |
| Creatinine (mg/dl)^5^ | >1.1 | Acute or chronic kidney disease |
| Blood urea nitrogen (mg/dl)^5^ | >20 |  |
| Total CO_2_ (mmol/L)^6^ | <20 | Respiratory and metabolic acidosis |
|  | >29 | Respiratory and metabolic alkalosis |
| Random blood sugar (mg/dL)^7^ | >200 | Diabetes |

^1^National Heart, Lung, and Blood Institute (NHLBI). Anemia - Diagnosis. Available from: <https://www.nhlbi.nih.gov/health/anemia/diagnosis.> Accessed 13 May 2024.

^2^ Kathak RR, Sumon AH, Molla NH, et al. The association between elevated lipid profile and liver enzymes: a study on Bangladeshi adults. Sci Rep 2022;12:1711.

^3^Institute of Medicine (IOM). Dietary reference intakes for calcium and vitamin D. Washington, DC: The National Academies Press; 2011.

^4^National Academies of Sciences, Engineering, and Medicine. Dietary reference intakes for sodium and potassium. Washington, DC: The National Academies Press; 2019.

^5^ Hosten AO. BUN and Creatinine. In: Walker HK, Hall WD, Hurst JW, editors. Clinical Methods: The History, Physical, and Laboratory Examinations. 3rd ed. Boston: Butterworths, 1990. Chapter 193. Available from: https://www.ncbi.nlm.nih.gov/books/NBK305/

^6^Hu J, Wang Y, Geng X, et al. Metabolic acidosis as a risk factor for the development of acute kidney injury and hospital mortality. Exp Ther Med 2017;13(5):2362-74. doi: 10.3892/etm.2017.4292 [published Online First: 20170330]

^7^American Diabetes Association (ADA). Diabetes Diagnosis & Tests. Available from: https://www.diabetes.org/diabetes/diagnosis. Accessed 13 May 2024.

Supplementary Figure 1. Management protocol of the weekly reporting of symptoms of side effects in MiNDR study


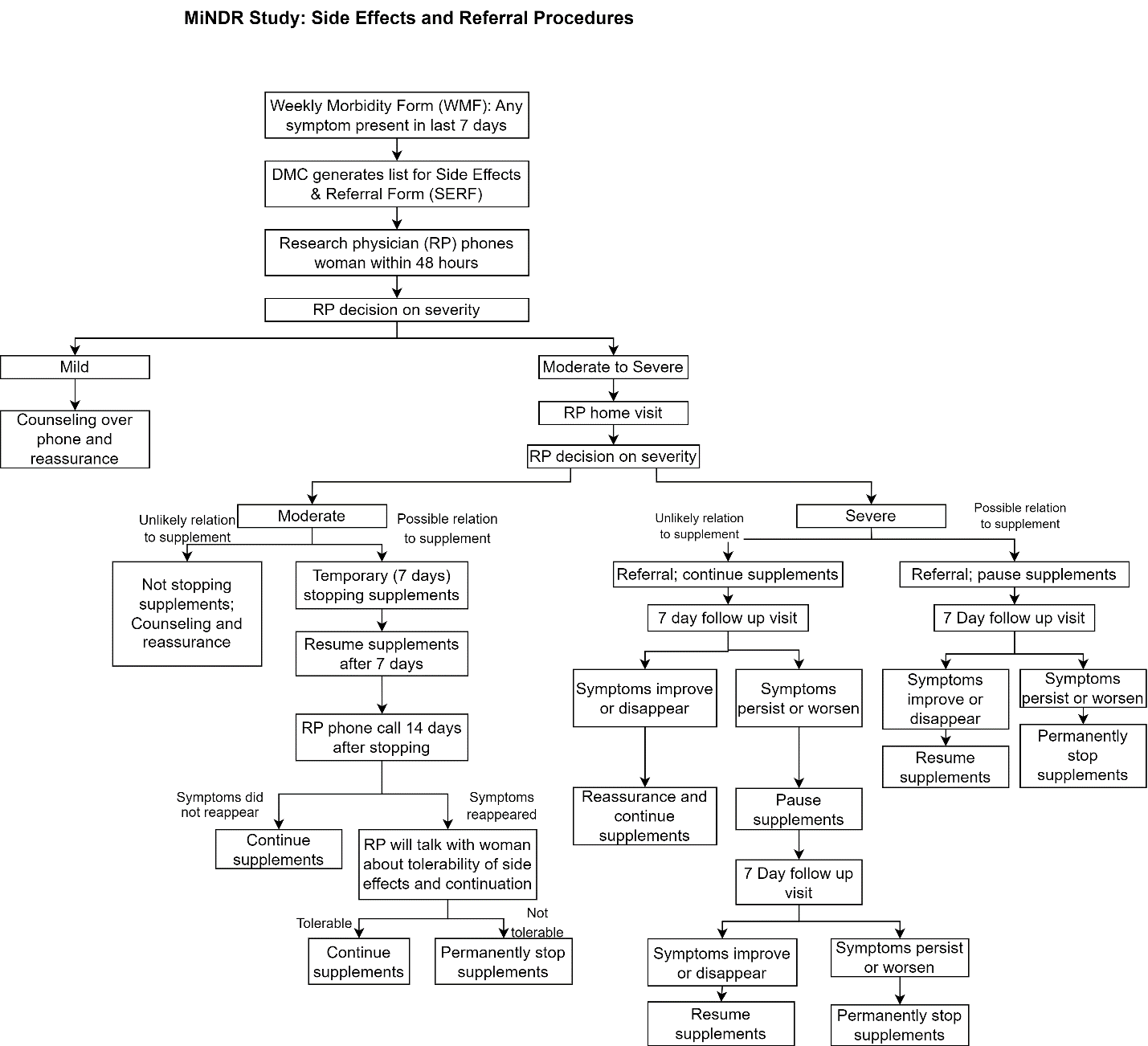

Supplement: online supplemental file 2 [file bmjopen-15-1-s002.docx]
