# Supplementary material for: Micronutrient dose response (MiNDR) study among women of reproductive age and pregnant women in rural Bangladesh: study protocol for double-blind, randomised, controlled trials
Source: BMJ Open. 2025 Jan 4;15(1):e090108. doi: 10.1136/bmjopen-2024-090108 (PMC11749533; doi:10.1136/bmjopen-2024-090108)
Supplement: online supplemental file 3 [file bmjopen-15-1-s003.pdf]

JOHNS HOPKINS BLOOMBERG SCHOOL OF PUBLIC HEALTH

ADULT INFORMED CONSENT
For
Women of Reproductive Age (WRA) Trial Enrollment

Principal Investigator: Dr. Parul Christian

Study Title: Micronutrient Dose-Response among Women of Reproductive Age and Pregnant Women in Rural Bangladesh: The MINDR Study.
IRB No.: 22171

Funded By: Bill & Melinda Gates Foundation

PI Version Date: Version 2, August 18, 2023

Key Information about the Study

Asalamualaikum. I am [name of female interviewer], a worker with the JiVitA project. As part of a JiVitA project we are conducting a new study collaboration with Johns Hopkins University and International Center for Diarrheal Disease Research, Bangladesh (ICDDR'B). We are asking you now to take part in a study in which we want to learn more about how much of different minerals and vitamins women of reproductive age should consume to maintain the best possible nutritional status. You are eligible to participate in this study because we understand that you are not intending to become pregnant for at least several months and/or are using an effective means of contraception. As part of this study, we will provide you with nutrients for 3 months in a food product in a packet and in a drink. We will measure vitamins and minerals and other things related to your health and well-being in your blood, urine, and stool at the beginning, middle, and end of the study. I would like to further explain the study and request your consent for participation, and I will answer any questions you have about the project.

You are free to choose to participate or not in this study and there is no penalty for not participating. Ask as many questions as you need to help you make your decision. There are few risks related to participating in this study but participating in the study may cause you some inconvenience or discomfort and some questions may seem sensitive. There is some risk to your privacy. There are no financial benefits or costs to participating other than the time you will spend speaking with me or other JiVitA workers.

Details about the Study

If you are willing to join, we will start today or another time soon at your convenience by asking you a variety of questions about your pregnancy history, illness, the food you ate yesterday (which we will ask about again later the same week), and some questions about your household belongings.

If you agree to join this study you will be assigned to receive a food product and a powder that contains one of 4 different nutrient levels for many of the nutrients your body needs. Which level you get will be determined by chance, like a lottery. You will not be able to tell which level of nutrients you receive, and neither will JiVitA workers, by looking at them. The powder containing nutrients will be made up into a drink and you will consume the food packet and take the drink daily for 3 months. You will take these products daily under the supervision of a JiVitA worker who will come by your house for seven days a week until the end of the supplementation period. On days you are not met at home, she will reach you by phone to make sure you are consuming these

products. Once a week she will ask you about how you feel, and she will also replace the products daily so that you don't run out of them.

You will be visited 3 times during the study by a team of JiVitA workers to measure height, weight, and arm circumference and collect blood, urine and stool. The first visit will happen before you receive your food packets and micronutrient powder, the second visit will happen one and a half months after you start taking the food and drink, and the third visit will happen three months after you start taking them—which will also be the end of the study. JiVitA workers will take body measurements of your height, weight, and arm circumference and will measure your hemoglobin and blood pressure. They will also collect about 2 spoonfuls of blood from your arm at each visit and a sample of urine in a cup. At the first and last visit they will also collect a stool sample. We will leave containers with you so that you can collect urine and stool privately. These specimens help us to understand how much of the nutrients in the food and drink product your body takes in.

Around the same time as those visits, JiVitA interviewers will also ask about any symptoms of illness you experienced in the last month, and at the end of the study they will also ask about your diet in the previous day at 2 different visits in the same week. Knowing more about what you eat can help us understand what nutrients you typically get from your diet alone.

The blood, urine, and stool will be taken to the JiVitA lab in Rangpur and samples will be shared with a laboratory at icddr,b in Dhaka and at Johns Hopkins University in the United States. Those labs will measure nutrients and other things that tell us about your nutritional and health status and whether the drink and food that you consumed changed them. We will not be able to share those results with you, although we will provide you with information about your blood group status and inform you if you are anemic. We will also have your stool sample analyzed to see what kind of bacteria are in your gut—some bacteria might affect how your body uses vitamins and minerals.

Not all the tests we might want to do on your biospecimens will be done right away, so we want permission to store some biospecimens for a long time so that we can do some tests on them later. For example, we might take some of the cells in your blood and look to see if the genes of study participants make the body use nutrients in a certain way or affect the body's ability to respond to nutrients in supplements. For any of the testing we do, now or later, your name and personal information will not be directly associated with the biospecimens you provide.

Why is this research being done?

This research is being done to provide information to the Bangladeshi government and other countries about the amounts of vitamins and minerals required by women, as well as during pregnancy to achieve good nutritional status and health.

By measuring the nutrients in your body and from other participants, we will be able to tell what levels work best for supporting women's health.

What will happen if you join this study?

There are several types of study visits that you'll be asked to complete if you join this study, and at least 3 different kinds of JiVitA workers who will visit you, over the 3 months of the study.

Enrollment Week Visits: If you agree to participate in the study, I will ask you some questions about your pregnancy/contraceptive history, recent illness, chronic health conditions, and about all the foods you ate

yesterday. I will also ask you about socioeconomic condition, the things you own and the number of years of schooling you have received. This interview will take about 40-45 minutes to complete. Later today, another JiVitA worker will visit to bring you containers and instructions for collecting stool and a urine sample, and another study team will visit you tomorrow, or as soon as possible.

Those workers will visit you to measure your height, weight, arm size and blood pressure, take a blood sample from your arm, and take your stool and urine specimens. We will measure hemoglobin to check if you have anemia, and we will share that information with you on the spot and provide you iron if you are severely anemic. This visit will take about 30 minutes. Also, a JiVitA Community Health Research Worker will visit you seven days a week to make sure you take the food and drink and will call you if you are not found at home to ask you if you took your drink and ate the food. They will also ask you about whether you have any symptoms or side effects. If some of the information we collect suggests that you have a reaction to the drink, we will send a physician to check on you and to refer you to a doctor for care, if needed. The daily visit will take about 15 minutes and the phone call about 5 minutes. If the blood tests suggest you have a health concern, a JiVitA physician will come to tell you what they have found, collect more information, and you may be referred for care and discontinued from the study.

<u>Follow-up Visits:</u> One and a half months from now and at 3 months JiVitA teams will come again to ask about recent illnesses, to measure your height, weight, arm size, and blood pressure, and to collect blood and urine. Stool will be collected only at the 3 month visit. Each of these visits will take about 30-40 minutes of your time.

If you agree to join this study, it is important that you:

- Agree to take daily both the food product and the drink every day.
- Agree to provide blood, urine and stool specimens.

What happens to data that are collected in the study?

The data we collect from you will be useful to increase our knowledge about nutrition and health of women. As a participant, you will not own your research data. Sharing of research data is often done to increase what scientists can learn. The data you provide us might be shared directly with Ethical Review Committees, other researchers, funders, government agencies, publishers of papers and through government or other databases/repositories

We will do our best to protect the data you provide and data will only be shared without your name, address, and date of birth. If data are shared with this information, further review and approval by an ethical committee might be required. If you are not comfortable with the use of your data in future research, we will not use this data in future studies.

What are the risks or discomforts of the study?

<u>Consumption of the vitamin and mineral powder in a drink:</u> Remembering to take this drink daily may cause some inconvenience. Sometimes women report having gut discomfort when they take supplements. We will ask you about this and other possible side effects of consuming the vitamins and minerals in the drink to make sure that they are not making you feel uncomfortable. We also ask that you not take any other supplements during the study.

<u>Consumption of the Food Product:</u> The food product made with puffed rice, lentil, oil and milk powder has been developed to meet the special nutritional needs of women living in rural areas. You may become bored of its

taste or texture, and it may be an inconvenience to finish a daily packet and to remember to eat it daily. Similar products have been tested and shown to be beneficial for women of reproductive age.

Measurements and Specimens Collection, Storage and Future Analysis:
Blood will be taken by well-trained JiVitA staff. Drawing blood will cause momentary pain and there is a small chance of a bruise, swelling or fainting. Blood will be drawn by trained staff who will follow all safety and sanitary procedures. There will be minimal risk of infection. There is no health risks of the urine and stool collection. Other measurements to be taken on you are safe.

Interviews and Questionnaires: You may get tired or bored while we ask you questions. Some questions may make you feel embarrassed or uncomfortable. Let us know if you feel distressed. You do not have to answer any question you do not want to answer.

Personal Privacy and Identifiable Private Information: There is a risk that information about you may become known to people outside this study. We will protect your information and conduct all interviews in private to reduce the chance of this happening.

How will the confidentiality of your data be protected?

Any information we collect and measurements we take will be kept confidential by JiVitA staff. Your identity will not be revealed when the information is used. Data will be stored securely at the JiVitA Data Management Center in Rangpur, at icddr,b, Dhaka, Bangladesh, and at Johns Hopkins University in Baltimore, Maryland, USA. Senior study staff and researchers at JiVitA, icddr,b, Johns Hopkins, and collaborating researchers will have access to the data. Whenever possible, we will remove all data that can identify you before sharing. This means removing your personal details like name and address. Once the study is completed, we will share data that is generated with the study sponsor. This will help researchers around the world understand how to best improve women's health and nutritional status. Information that identifies you specifically (eg, your name or the name of anyone in your household and location of your household) will be removed from the database before any data is shared.

What are the potential benefits to being in the study?

There may be some health benefits to you from participating in this trial. As I mentioned earlier, consuming a nutritional drink and food product could benefit you. When we test your blood we will let you know your blood grouping result in the spot. We will also check your hemoglobin. If you are severely anemic, we will provide you iron tablets free of cost and refer you for further care if needed. If you have high blood pressure or if we notice other health issues, we will refer you to a doctor for care. There is no direct benefit to you in allowing us to store your samples. However, the use of your samples in future research could help us learn more about the health of women and how to improve it. Additionally, you will be given a token gift to thank you for your participation in this study. We will also provide snacks or a drink after the blood draw to make sure that you feel okay.

Will you be paid if you join this study?

We will not pay you for participating in the study, other than with a small gift. We will make sure that we conduct study visits at a time that is convenient for you. In the event you experience any study-related injuries, we will provide a referral to seek appropriate care. All costs associated with the referral and management will be borne by the project.

Can you leave the study early?

Your decision to participate in this study is up to you. You may stop participating in the research study at any time. You can agree now and change your mind later. If you leave the study early, researchers may use or share your health information that has already been collected, if the information is needed for this study or any follow-up activities.

What other things should you know about this research study?

A description of this effectiveness study will be available on http://www.ClinicalTrials.gov as required by U.S. Law. This Web site will not include information that can identify you. At most, the Web site will include a summary of the results. You can search this Web site at any time.
If you would like to review the information for this study, or a summary of the results, ask the study team doctor for the ClinicalTrials.gov study registration number.

What is the Institutional Review Board (IRB) and how does it protect you?

This study has been reviewed by an Institutional Review Board (IRB), a group of people including scientists and community people that reviews human research studies. The IRB can help you if you have questions about your rights as a research participant or if you have other questions, concerns or complaints about this research study. You may contact the icddr,b IRB at 01711428989 or salamk@icddrb.org

What should you do if you have questions about the study, or are injured or ill as a result of being in this study?

Call the Senior Project Investigators, Dr. Towfida Siddiqua (Mobile No. 01713202558) or Dr. Hasmot Ali (Mobile No. 01713163461). If you wish, you may contact the principal investigator by letter:
Faijun Villa, Road No 01, House No. 03
Nasirabad, Kerani Para, Rangpur-5400, Bangladesh.

What does your signature/thumb print on this consent form mean?

Your signature on this form means that you have reviewed the information in this form, you have had a chance to ask questions, and you agree to join the study. You will not give up any legal rights by signing this consent form.

WE WILL GIVE YOU A COPY OF THIS SIGNED AND DATED CONSENT FORM

Thank you for your co-operation.

____
Signature/Thumb print of Participant (Print Name) Date/Time

____
Signature of Person Obtaining Consent (Print Name) Date/Time

Worker Use Only:
Sign and Date two copies of consent form. Complete Consent Status Codes Below and Copy to [ROSTER]

Participant Name: ___________________ Participant UID: __ __ __ __ __ __

☐ Consent to participate in the study

Consent Status Codes:
1=Yes, Agreed

6=No, Refused

☐ Consent to sharing data

Consent Status Codes:
1=Yes, Agreed
6=No, Refused

☐ Consent to store biospecimens for future analysis

Consent Status Codes:
1=Yes, Agreed
6=No, Refused
