# Supplementary material for: Micronutrient dose response (MiNDR) study among women of reproductive age and pregnant women in rural Bangladesh: study protocol for double-blind, randomised, controlled trials
Source: BMJ Open. 2025 Jan 4;15(1):e090108. doi: 10.1136/bmjopen-2024-090108 (PMC11749533; doi:10.1136/bmjopen-2024-090108)
Supplement: online supplemental file 4 [file bmjopen-15-1-s004.pdf]

JOHNS HOPKINS BLOOMBERG SCHOOL OF PUBLIC HEALTH

ADULT INFORMED CONSENT
for
Pregnant Women Trial Enrollment

Principal Investigator: Dr. Parul Christian

Study Title: Micronutrient Dose-Response among Women of Reproductive Age and Pregnant Women in Rural Bangladesh: The MINDR Study

IRB No.: 22171

Sponsor/Supporter/Funded By: *Bill & Melinda Gates Foundation*

PI Version Date: Version 2, August 18, 2023

Key Information about the Study

Assalamualaikum. I am [name of female interviewer], a worker with JiVitA project. As part of a JiVitA project we are conducting a new study collaboration with Johns Hopkins University and International Center for Diarrheal Disease Research, Bangladesh (icddr,b). We are asking you now to take part in the study where we want to learn more about how much of several nutrients pregnant women need to maintain the best possible nutritional status. You are eligible to participate in this study because you just had a positive pregnancy test. As part of this study, we will provide you with nutrients for about 6 months in a food product in a packet and a drink. We will measure vitamins and minerals and other things related to your health and well-being in your blood, urine, and stool during your pregnancy and in blood, urine, and your breast milk a month after you give birth.

I would like to explain the study and request your consent for participation, and I will answer any questions you have about the project.

You are free to choose to participate or not in this study and there is no penalty for not participating. Ask as many questions as you need to help you make your decision. There are few risks related to participating in this study but participating in the study may cause you some inconvenience or discomfort, and some questions may seem sensitive. There is some risk to your privacy. There are no financial benefits or costs to participating other than the time you will spend speaking with me or other JiVitA workers.

Details about the Study

If you are willing to join, we will start today or another time soon at your convenience by asking you a variety of questions about your pregnancy history, illness, the food you ate yesterday (which we will ask about again later the same week), and some questions about your household belongings.

If you agree to join this study, you will be assigned to receive a food product and a powder in a sachet that contains one of 4 different nutrient levels for many of the nutrients your body needs. The powder with the lowest levels of nutrients will be equivalent to the current amounts recommended for pregnant women. Which level you get will be determined by chance, like a lottery. You will not be able to tell which level of nutrients you receive, and neither will JiVitA workers, by looking at them. The powder containing nutrients will be made up into a drink. You will take these products daily under the supervision of a JiVitA worker who will come by your

house seven days a week until the end of pregnancy. On days you are not met at home, she will reach you by phone to make sure you are consuming these products. Once a week she will ask you about how you feel, and she will also replace the products daily so that you don't run out of them.

You will be visited 5 times during the study by a team of JiVitA workers to measure height, weight, and arm circumference and collect blood, urine, stool and breastmilk. The first visit will be for an ultrasound measurement, so that we can tell how long you have been pregnant. At the next visit, JiVitA workers will take body measurements of your height, weight, and arm circumference and will measure your hemoglobin and blood pressure. They will also collect about 2 spoonfuls of blood from your arm and a sample of urine in a cup. Those procedures will be repeated in the middle and end of your pregnancy, and one month after you give birth. At the first and late pregnancy visit they will also collect a stool sample, and at the visit after delivery they will collect a sample of breast milk. We will leave a container with you so that you can collect urine and stool privately. To collect breast milk we will ask you to pump all the milk out of one breast, but we will only collect a small amount of it and will return the rest to you to feed to your baby. All these specimens help us to understand how much of the nutrients in the food and drink product your body takes in. Finally, shortly after your baby's birth we will check in on you and your baby, ask some questions about your labor and delivery experience, and measure your baby's weight and length.

At the visits, JiVitA interviewers will also ask about any symptoms of illness you experienced in the last month and will also ask about your diet in the previous day at 2 different visits in the same week. Knowing more about what you eat can help us understand what nutrients you typically get from your diet alone.

The blood, urine, stool, and milk that are collected will be taken to the JiVitA lab in Rangpur, and samples will be shared with a laboratory at icddr,b in Dhaka and at Johns Hopkins University in the United States. Those labs will measure nutrients and other things that tell us about your nutritional and health status and whether the special drink and food that you consumed changed them. We will not be able to share those results with you, although we will provide you with information about your blood group status and inform you if you are anemic. We will also have your stool sample analyzed to see what kind of bacteria are in your gut—some bacteria might affect how your body uses vitamins and minerals.

Not all the tests we might want to do on your biospecimens will be done right away, so we want permission to store some biospecimens for a long time so that we can do some tests on them later. For example, we might take some of the cells in your blood and look to see if the genes of study participants make the body use nutrients in a certain way or affect the body's ability to respond to nutrients in supplements. For any of the testing we do, now or later, your name and personal information will not be directly associated with the biospecimens you provide.

Why is this research being done?

This research is being done to provide information to the Bangladeshi government and other countries about the amounts of vitamins and minerals required by women, as well as during pregnancy to achieve good nutritional status and health.

By measuring the nutrients in your body and from other participants, we will be able to tell what levels work best for supporting women's health.

What will happen if you join this study?

There are several types of study visits that you'll be asked to complete if you join this study. There are three different kinds of JiVitA workers who will visit you over the 6-7 months of the study.

- **Enrolment Week Visits:** If you agree to participate in the study, I will ask you some questions about your pregnancy/contraceptive history, recent illness, chronic health conditions, and all the foods you ate yesterday. We will also ask you about socioeconomic condition, the things you own and the number of years of schooling you have received. This visit will take about 40-45 minutes to complete. Later today, JiVitA workers will visit you to provide you with containers and instructions for collecting stool and urine samples, and they will return to pick up the samples on the following day.
    - A JiVitA staff member will come to do an ultrasound exam to determine the duration of your pregnancy. This visit will take about 20-25 minutes.
- Another team will visit you to measure your height, weight, arm size and blood pressure. Additionally, a trained nurse will collect blood (about 2 small spoons) into a tube from your arm. We will measure hemoglobin to check if you have anemia, and we will share that information with you on the spot and provide you iron if you have severe anemia for free. This visit will take about 30 minutes. Also, a JiVitA Community Health Research Worker will visit you seven days a week to make sure you take the food and drink and call you if you are not found at home to ask you if you took your drink and ate the food. They will also ask you about whether you have any symptoms or side effects. If some of the information we collect suggests that you have a reaction to the drink, we will send a physician to check on you and to refer you to a doctor for care, if needed. The daily visit will take about 15 minutes and the phone call about 5 minutes. If the blood tests suggest you have a health concern, a JiVitA physician will come to tell you what they have found, collect more information, and you may be referred for care and discontinued from the study.
- **Mid and late pregnancy visits:** At 22 or 30 weeks of gestation, we will visit your household to ask some questions about your health and food that you have eaten in last 24 hours, measure your weight, size of your arms, blood pressure, hemoglobin and collect blood and urine samples. Later in pregnancy at 36 weeks of gestation, we will visit your household to ask the same questions and measurements as well as collect blood, urine, and stool samples. Interview and specimen collection procedures will be similar as enrolment visit. This visit will take 30 to 40 minutes.
- **Birth Visit:** We will visit you soon after delivery to ask questions about labor and delivery. We will measure your weight, size of your arms as well as your baby's weight, length, head, arm, and chest circumference.
- **1-month postpartum visit**: One month after birth, we will visit you for the last time. We will measure your weight, size of your arm, hemoglobin, blood pressure, and ask questions about your health since your baby was born as well as about your diet in the last 24 hours. We will also ask you about how your baby is doing and about breastfeeding and early feeding and will take your baby's weight, length, head, arm, and chest circumference. We will collect your blood, urine and breast milk samples at this time point. This visit will take about 45 minutes of your time.

If you agree to join this study, it is important that you:

- Agree to take daily both the food product and the drink every day.
- Agree to provide blood, urine, stool and breastmilk specimens.

What happens to data that are collected in the study?

The data we collect from you will be useful to increase our knowledge about nutrition and care pregnant women need. As a participant, you will not own your research data, and you will not benefit financially from any new product or idea that might arise from our work. Sharing of research data is often done to increase what scientists can learn. The data you provide us might be shared directly with Ethical Review Committees, other researchers, funders, government agencies, publishers of papers and through government or other databases/repositories. We will do our best to protect the data you provide and data will only be shared without your name, address, and date of birth. If data are shared with this information, further review and approval by an ethical committee might be required. If you are not comfortable with the use of your data in future research, we will not use this data in future studies.

What are the risks or discomforts of the study?

Consumption of the vitamin and mineral powder in a drink: Remembering to take this drink daily may cause some inconvenience. Sometimes women report having gut discomfort when they take supplements. We will ask you about this and other possible side effects of consuming the vitamins and minerals in the drink to make sure that they are not making you feel uncomfortable. We also ask that you not take any other supplements during the study.

Consumption of the Food Product: The food product made with puffed rice, lentil, oil and milk powder has been developed to meet the special nutritional needs of women living in rural areas. You may become bored of its taste or texture and it may be an inconvenience to finish a daily packet and to remember to eat it daily. Similar products have been tested and shown to be beneficial for women of reproductive age.

Measurements and Specimens Collection, Storage and Future Analysis: Blood will be taken by well-trained JiVitA staff. Drawing blood will cause momentary pain and there is a small chance of a bruise, swelling or fainting. Blood will be drawn by trained staff who will follow all safety and sanitary procedures. There will be minimal risk of infection. There are no health risks of the urine, breastmilk and stool collection. Other measurements to be taken on you are safe.

Interviews and Questionnaires: You may get tired or bored while we ask you questions. Some questions may make you feel embarrassed or uncomfortable. Let us know if you feel distressed. You do not have to answer any question you do not want to answer.

Personal Privacy and Identifiable Private Information: There is a risk that information about you may become known to people outside this study. We will protect your information and conduct all interviews in private to reduce the chance of this happening.

How will the confidentiality of your data be protected?

Any information we collect and measurements we take will be kept confidential by JiVitA staff. Your identity will not be revealed when the information is used. Data will be stored securely at the JiVitA Data Management Center in Rangpur, at icddr,b, Dhaka, Bangladesh, and at Johns Hopkins University in Baltimore, Maryland, USA. Senior study staff and researchers at JiVitA, icddr,b, Johns Hopkins, and collaborating researchers will have access to the data. Whenever possible, we will remove all data that can identify you before sharing. This means removing your personal details like name and address. Once the study is completed, we will share data that is generated with the study sponsor. This will help researchers

around the world understand how to best improve women's health and nutritional status. Information that identifies you specifically (eg, your name or the name of anyone in your household and location of your household) will be removed from the database before any data is shared.

What are the potential benefits to being in the study?

There may be some health benefits to you from participating in this study. As I mentioned earlier, consuming a nutritional drink and food product could benefit you. When we test your blood we will let you know your blood grouping result in the spot. We will also check your hemoglobin. If you are severely anemic, we will provide you iron capsules free of cost and refer you for further care, if needed. If you have high blood pressure or if we notice other health issues, we will refer you to a doctor for care. Moreover, by doing ultrasonography, you will be able to know your pregnancy status more accurately. There is no direct benefit to you in allowing us to store your samples. The use of your samples in future research could help us learn more about the health of women and how to improve it. Additionally, you will be given a token gift to thank you for your participation in this study. We will also provide snacks or a drink after the blood draw to make sure you feel okay.

Will you be paid if you join this study?

There is no cost to you for participating in the study, other than with a small gift. We will make sure that we conduct study visits at a time that is convenient for you. In the event you experience any study-related injuries, we will provide a referral to seek appropriate care. All costs associated with the referral and management will be borne by the project.

Can you leave the study early?

Your decision to participate in this study is up to you. You may stop participating in the research study at any time. You can agree now and change your mind later. If you leave the study early, researchers may use or share your health information that it has already collected if the information is needed for this study or any follow-up activities.

What other things should you know about this research study?

A description of this effectiveness study will be available on http://www.ClinicalTrials.gov, as required by U.S. Law. This Web site will not include information that can identify you. At most, the Web site will include a summary of the results. You can search this Web site at any time.
If you would like to review the information for this study, or a summary of the results, ask the study team doctor for the ClinicalTrials.gov study registration number.

<u>What is the Institutional Review Board (IRB) and how does it protect you?</u>

This study has been reviewed by an Institutional Review Board (IRB), a group of people including scientists and community people, that reviews human research studies. The IRB can help you if you have questions about your rights as a research participant or if you have other questions, concerns or complaints about this research study. You may contact the ICDDR'B IRB at 01711428989or salamk@icddrb.org

What should you do if you have questions about the study, or are injured or ill as a result of being in this study?

Call the Senior Project Investigators, Dr. Towfida Siddiqua (Mobile No. 01713202558) or Dr. Hasmot Ali (Mobile No. 01713163461). If you wish, you may contact the principal investigator by letter:

Faijun Villa, Road No 01, House No. 03, Nasirabad, Kerani Para, Rangpur-5400, Bangladesh.

What does your signature/thumb print on this consent form mean?

Your signature on this form means that you have reviewed the information in this form, you have had a chance to ask questions, and you agree to join the study. You will not give up any legal rights by signing this consent form.

WE WILL GIVE YOU A COPY OF THIS SIGNED AND DATED CONSENT FORM

Thank you for your co-operation.

____

Signature/Thumb print of Participant (Print Name) Date/Time

____

Signature of Person Obtaining Consent (Print Name) Date/Time

Worker Use Only:

Sign and Date two copies of consent form. Complete Consent Status Codes Below and Copy to [ROSTER]

Participant Name: __________________ Participant UID: __ __ __ __ __ __

☐ Consent to participate in the study

Consent Status Codes:

1=Yes, Agreed

6=No, Refused

☐ Consent to sharing data

Consent Status Codes:

1=Yes, Agreed

6=No, Refused

☐ Consent to store biospecimens for future analysis

Consent Status Codes:

1=Yes, Agreed

6=No, Refused
