# Supplementary material for: Micronutrient dose response (MiNDR) study among women of reproductive age and pregnant women in rural Bangladesh: study protocol for double-blind, randomised, controlled trials
Source: BMJ Open. 2025 Jan 4;15(1):e090108. doi: 10.1136/bmjopen-2024-090108 (PMC11749533; doi:10.1136/bmjopen-2024-090108)
Supplement: Supplementary file 5 [file bmjopen-2024-090108.reviewer_comments.pdf]

PEER REVIEW HISTORY

BMJ Open publishes all reviews undertaken for accepted manuscripts. Reviewers are asked to complete a checklist review form and are provided with free text boxes to elaborate on their assessment. These free text comments are reproduced below.

ARTICLE DETAILS

Title (Provisional)

Micronutrient Dose Response (MiNDR) Study among Women of Reproductive Age and Pregnant Women in Rural Bangladesh: Study Protocol for Double-blind, Randomized, Controlled Trials

Authors

Siddiqua, Towfida Jahan; Schulze, Kerry J; Hasan, S. M. Tafsir; Ahsan, Khalid Bin; Bandyopadhyay, Sulagna; Zavala, Eleonor; Ali, Hasmot; Haque, Rezwanul; Sujan, Hasan Mahmud; Rahman, Md. Hafizur; Baker, Sarah; Stephenson, Katherine K.; Ge, Ximing; Gough, Ethan K.; Langevin, Brooke Ashley; Wu, Lee Shu Fune; Dyer, Brian; Roy, Anjan Kumar; Jubair, Mohammad; Nishan, Amena Al; Rosenblum, Michael; Gopalakrishnan, Mathangi; Kraemer, Klaus; Erchick, Daniel J.; Ahmed, Tahmeed; Christian, Parul

---

VERSION 1 - REVIEW

**Reviewer**	**1**
**Name**	**Mayasari, Noor Rohmah**
**Affiliation**	**State University of Surabaya, Nutrition**
**Date**	**17-Jul-2024**
**COI**	**I declare no competing interest**

---

1. Subject pregnant women and women of reproductive age. Will the analysis combine this two-population population?

2. Could you specify interventions A, B, C, and D, and which is the placebo?

3. Would you consider Dietary intake during the study as a confounding factor?

**Reviewer**	**2**
**Name**	**van Klinken, Bernardus**
**Affiliation**	**Brightseed, consumer hc**
**Date**	**22-Oct-2024**

COI **none**

This is important researching population groups that have been under-researched and should deliver critically important information to optimize MN supplementation strategies and provide input to developing recommended intake levels. The outcome will enable important insights for further studies and allow for policy recommendations in this underserved population. It is well written and clear.

A few comments:

1. The hypothesis and objectives could be worded more clearly. i.e. what is hypothesized on response in MN status of PW vs. WRA? will PW have higher nutrient requirements or take longer to note significant change? Is 3 months not too short to see an appreciable change in MN status in the WRA group? Why 6 months in PW and not in WRA?

2. A limitation in the power analysis is that effect size of MN status improvement vs,. supplied quantity of MN is unknown as stated by the authors. Therefore such a power analyses on expected effect size (increase in MN status vs. dose) could not be calculated. Obviously the outcome of this study should clarify this area but not knowing this at the start of the study is a limitation and should be stated. Note that effect size of B12 supplementation in PW in a similar population was previously reported by this research group [Siddiquia et al, 2015 EJN]

One suggestion to include in the biomarker analysis also DNA adduct formation, telemore length, Micronuclei index and other measures of chromosomal aberrations. MN status affects these biomarkers and they may prove to be effective indicators. I would be happy to refer the researchers. Please see Fenech MF et al. .Adv Nutr. 2023 Nov;14(6):1337-1358. DOI: 10.1016/j.advnut.2023.08.004

VERSION 1 - AUTHOR RESPONSE

Reviewer: 1 **(Dr. Noor Rohmah Mayasari, State University of Surabaya)**

Comment 1: Subject pregnant women and women of reproductive age. Will the analysis combine this two-population population?

> **Response:** We are conducting two parallel dose-response trials, one in pregnant women and one in women of reproductive age; therefore, data analysis will be performed separately for each trial. We have now included a sentence in the data analysis section to clarify this (Page 25, Line 490).

Comment 2: Could you specify interventions A, B, C, and D, and which is the placebo?

> **Response:** In these double-blind, randomized controlled trials involving pregnant women and women of reproductive age, the interventions, specifically the nutrient supplements provided in the form of a powdered reconstituted drink, are identical in appearance and labeled with codes A, B, C, and D, one of which is a placebo. The codes are securely stored in a sealed envelope at a central location managed by the

product manufacturer and by an independent senior staff from the collaborating institution in Bangladesh (icddr,b). The blinding of the investigation teams, participants, data collectors and lab and data analysts will be maintained until the end of the study, as detailed on Page 11, Lines 182-185.

Comment 3: Would you consider Dietary intake during the study as a confounding factor?

Response: It may be a confounder or an effect modifier. We are collecting dietary information from all participants using the multiple-pass 24-hour recall method at enrollment and during subsequent follow-up visits, from which the usual intake of nutrients will be calculated. We will add analyses that adjust for differential intakes of specific nutrients in examining dose response to the supplement.

Reviewer: 2 (Dr. Bernardus van Klinken, Brightseed, NutritioMed)
This is important researching population groups that have been under-researched and should deliver critically important information to optimize MN supplementation strategies and provide input to developing recommended intake levels. The outcome will enable important insights for further studies and allow for policy recommendations in this underserved population. It is well written and clear.

Response: We thank the reviewer for appreciating our work and the manuscript.

Comment 1: The hypothesis and objectives could be worded more clearly. i.e. what is hypothesized on response in MN status of PW vs. WRA? will PW have higher nutrient requirements or take longer to note significant change? Is 3 months not too short to see an appreciable change in MN status in the WRA group? Why 6 months in PW and not in WRA?

Response: Thank you for pointing out that the rationale for our decisions around duration of supplementation were not clear. We have added details around the selection of the time periods for supplementation for the respective trials on pages 15-16, lines 275-276, and 283-286. As indicated there, we chose the 3-month timeframe for the WRA trial based on input from a technical advisory group. For most nutrients, including minerals, this is a reasonable period in which to expect changes in the conventional nutritional biomarkers of status. For the PW trial, the intent was to be consistent with the duration of time that women would receive supplements during pregnancy for the duration of second and third trimester, once they know their pregnancy status. We do believe that the burden of micronutrient deficiencies will be greater among PW in the absence of supplementation, given the enhanced nutritional demands of pregnancy.

Comment 2: A limitation in the power analysis is that effect size of MN status improvement vs,. supplied quantity of MN is unknown as stated by the authors. Therefore such a power analyses on expected effect size (increase in MN status vs. dose) could not be calculated. Obviously the outcome of this study should clarify this area but not knowing this at the start of the study is a limitation and should be stated. Note that effect size of B12 supplementation in PW in a similar population was previously reported by this research group [Siddiquia et al, 2015 EJN].

Response: It is true that in trials testing the efficacy of a supplement or any other intervention the effect size is hypothesized based on the best available existing data and knowledge, and the sample size estimate is derived using such a data-based approach. As such we were able to use biomarker data from a trial (JiVitA-3; West KP et al, JAMA 2014;312:2649-58, Schulze et al, J Nutr 2019;149:1260-70) where antenatal supplementation with ~1RDA of a variety of nutrients was tested against the

standard of care of iron and folic-acid alone in the setting in which the current set of trials is being done. Thus, we have a fairly strong basis for understanding the extent to which micronutrient status will be affected per RDA increase in intake across a variety of micronutrients and given the expected variability of micronutrient biomarkers. This information was used to justify the sample size, such that the pharmacokinetic parameter of interest for the micronutrient (ie., clearance) can be precisely estimated. We have now referred to this data source more clearly on page 14, lines 237-239.

The single-nutrient study mentioned by the reviewer by Siddiqua et al provided nearly 100x the RDA, considered a pharmacologic dose, of vitamin B12 as part of an experimental trial, with a call for additional dosing studies. Another single-nutrient trial in India provided ~20x the RDA (50 µg) and found it improved total circulating B12 but did not affect other B12 biomarkers (Duggan C et al, J Nutr 2020; 144: 758-64). Another study showed poor bioavailability of vitamin B12 at ~6x the RDA (e.g., Devi S et al, Am J Clin Nutr 2020; 112:1504-15) compared to at the RDA. Our intent was to keep nutrient doses in ranges that would likely ultimately be considered for a multiple micronutrient supplement product for regular use rather than at levels considered pharmacologic. These considerations provide an example of how we triangulated information from the literature and our own research to choose relevant nutrient doses.

Comment 3: One suggestion to include in the biomarker analysis also DNA adduct formation, telemore length, Micronuclei index and other measures of chromosomal aberrations. MN status affects these biomarkers and they may prove to be effective indicators. I would be happy to refer the researchers. Please see Fenech MF et al. .Adv Nutr. 2023 Nov;14(6):1337-1358. DOI: 10.1016/j.advnut.2023.08.004

Response: It would be our great pleasure to collaborate with Dr. Fenech or others to further explore the data and biospecimens we will be collecting. As it happens, we have a friendly (albeit long-distance) relationship with Dr. Fenech. However, at this juncture these additional analyses are beyond the scope of our budget and current aims. Nonetheless, we look forward to conducting further analyses in the future, and this is an excellent suggestion.
